# Case of Empyema on the Right Side, Acute Pleurisy on the Left, Simulating Pericarditis

**Published:** 1855-09

**Authors:** John T. Metcalfe

**Affiliations:** 34 East Thirteenth street


*Case of Empyema on the right side, Acute Pleurisy on the left, simulating Pericarditis.* By JOHN T. METCALFE, M. D.—John Armstrong, æt. 23, chairmaker, entered the New York Hospital Jan. 30th, 1855. One week before admission, had been taken with a cough, pain in the right chest, and dyspnœa. At the same time, there was observed a loss of appetite, a general febrile reaction, thirst and headache. The cough was accompanied by scanty expectoration of mucus, not characteristic in appearance. Since the commencement of his attack, these symptoms have all diminished with the exception of the stitch-like pain in the side, of which he complains a good deal. On admission, the pulse was 96; respiration 36; tongue moist, coated; eyes suffused; general appearance of distress; lies most comfortably on left side. The whole of the right chest dull on percussion; no vesicular murmur audible on this side; bronchial respiration (deep) and broncophony. On left side, nothing abnormal, except puerile respiration generally diffused, detrusion of heart's apex one inch to the left. *Wet cups to affected side. Mixture of Spirits of Mindererus and Ipecac. To take every four hours, a pill of calomel, squill, and digitalis (one grain of each).*

Under this treatment, with a blister to the side, there was evident amendment, so far as regarded the pain and dyspnœa. The chest seemed to become more distended however, the heart's apex being displaced as low as the sixth intercostal space, four inches to the left of the sternum. In place of the bronchial sounds audible on entrance, there was absence of respiration, except at the very summit of the right lung. The patient was able to get up and walk about the ward, but his appearance was far from satisfactory.

*February* 14. Just after having eaten dinner, he was suddenly seized with very great dyspnœa, the respiration ran up to 60 in a minute and the pulse to 120. The skin was cold and clammy; the eyes were anxious and protruding; there was complaint of pain on the left side, over the præcordial and epigastric regions. Breathing could only be performed whilst the patient was held up in bed. The left chest seemed alone engaged in respiration. Over the præcordial region, in its lower and outer half, a strong, well marked fremitus, synchronous with the cardiac systole, was noticed on palpation. Where this existed, a rough friction murmur with the first sound of the heart, not transmitted along the aortic branches, was noted. At the upper portion of right chest, under the clavicle, were resonant percussion, amphoric respiration, and metallic tinkling, very well marked. An anodyne was ordered, of the *compound spirit of sulphuric ether with the solution of sulphate of morphia;* and directions given to let me know in case relief should not be obtained, so that paracentesis of the chest should be performed. He continued with great oppression of breathing, and in the course of the night died, somewhat suddenly.

On post-mortem examination, the right lung was found compressed to a size of two fists, and forced back against the spine at the upper part of the pleural sac. On inflation through the main bronchus, air escaped by several small perforations on the upper surface of the lung. These perforations occurred in the wall of a tuberculous cavity of considerable size, which had been reduced, by the pressure of surrounding fluid, to very small dimensions. Fibrous exudation covered the outer surface of the lung. The pleura was nearly quite full of cream-like, inodorous pus.

In the left pleural cavity were several ounces of recent serous exudation, in which floated shreds of false membrane. The outer surface of the lung was roughened by this exudation, especially that part which lay over the heart, in contact with the costal pleura. The lung tissue contained a few scattered tubercles. Beyond slight enlargement of the heart, there was nothing noticed abnormal with respect to it or to the pericardium.

This case was one in which the dyspnœa had been explained, during life, by the perforation of the lung and production of pneumothorax. With regard to the friction-sound and fremitus, existing as they very markedly did over the præcordial region only, and synchronous with the systole of the heart, no one who saw the case for a moment doubted the presence of pericarditis as the cause of their production. Pleurisy so situated and giving rise to these signs, is of extremely rare occurrence. In my experience, I remember only one other case in which a similar error in diagnosis has occurred. In this there was fibrinous exudation over the pulmonary pleura, which is in direct contact with the pericardium.

The patient had been carefully examined by the House Physician with reference to his previous state of health, but gave no account of any illness connected with the tubercular excavation in the right lung. His intelligence was not of a high order; and every one familiar with the interrogation of a majority of those admitted to the hospitals, will understand the difficulty of getting a full and truthful account of their previous complaints.

34 *East Thirteenth street.* *N. Y. Med. Times.*

